# Effect of ticagrelor versus clopidogrel on platelet reactivity measured by thrombelastography in patients with minor stroke or TIA

**DOI:** 10.18632/aging.103452

**Published:** 2020-10-16

**Authors:** Yingying Yang, Weiqi Chen, Yuesong Pan, Hongyi Yan, Xia Meng, Liping Liu, Yongjun Wang, Yilong Wang

**Affiliations:** 1 Department of Neurology, China National Clinical Research Center for Neurological Diseases, Beijing Tiantan Hospital, Capital Medical University, Beijing, China; 2 Advanced Innovation Center for Human Brain Protection, Capital Medical University, Beijing, China

**Keywords:** clopidogrel, platelet reactivity, stroke, ticagrelor, thrombelastography

## Abstract

In this study, we tested the effect of ticagrelor versus clopidogrel on platelet reactivity in patients with minor stroke or transient ischemic attack (TIA). A pre-specified subgroup analysis of a randomized controlled trial was conducted. Platelet reactivity was assessed by thrombelastography (TEG) platelet mapping. Patients were divided into carriers and non-carriers according to the carrier status of *CYP2C19* loss-of-function (LOF) alleles. The primary outcome was the proportion of patients with high on-treatment platelet reactivity (HOPR) (defined as maximum amplitude induced by adenosine diphosphate > 47mm) at 90±7 days. Clinical outcomes within 90±7 days were followed up. Among 339 patients, 170 were randomized to ticagrelor/aspirin and 169 to clopidogrel/aspirin. Compared with clopidogrel/aspirin, the proportion of HOPR at 90±7 days in ticagrelor/aspirin was significantly lower (12.2% versus 30.0%, *P* < 0.001). Ticagrelor/aspirin had a lower proportion of HOPR among carriers (11.0% versus 35.6%, *P* < 0.001), but not among non-carriers (13.5% versus 22.4%, *P* = 0.17). Ticagrelor was superior to clopidogrel in inhibiting platelet reactivity measured by TEG platelet mapping among patients with acute minor stroke or TIA, particularly in carriers of the *CYP2C19* LOF alleles. Large randomised controlled trials are needed to confirm our findings.

## INTRODUCTION

The risk of recurrent stroke and other vascular events is high after acute minor stroke and high-risk transient ischemic attack (TIA) (5-11.7%) [1–3]. Dual antiplatelet therapy, combination of clopidogrel and aspirin, is an effective strategy for reducing recurrence [4–6]. However, clopidogrel resistance occurs in 5-30% of patients [7] and the *CYP2C19* loss-of-function (LOF) alleles are related to poor clopidogrel metabolism [8–10].

Unlike clopidogrel, the metabolism of ticagrelor is primarily via the CYP3A4 enzyme and does not involve *CYP2C19* [11] and ticagrelor inhibits platelet reactivity irrespective of *CYP2C19* genotypes [12, 13]. Previous studies have shown that ticagrelor is more effective than clopidogrel in inhibiting platelet reactivity and reducing the recurrence of ischemic vascular events in patients with acute coronary syndrome [14, 15]. However, limited data are available from patients with cerebrovascular diseases, and whether treatment with ticagrelor is superior to clopidogrel remains unclear.

Platelet function tests can evaluate the response variability to antiplatelet therapies. High on-treatment platelet reactivity (HOPR) is associated with the risk of ischemic events, and reflects a poor response to antiplatelet therapies [16, 17]. Platelet Reactivity in Acute Non-disabling Cerebrovascular Events (PRINCE) trial showed that ticagrelor had a lower proportion of HOPR assessed by VerifyNow P2Y12 assay than clopidogrel in patients with minor stroke and TIA [18]. Thrombelastography (TEG), a fast and efficient whole-blood test with high specificity, can not only assess coagulation and fibrinolysis process, but also be used to predict the risk of both thrombosis and bleeding [19]. TEG is widely used for the measurement of platelet function, particularly among patients with percutaneous coronary intervention [20–28]. However, few studies have investigated the application of TEG in minor stroke and TIA. In this pre-specified subgroup analysis of PRINCE trial, we aimed to assess the comparative effects of ticagrelor versus clopidogrel on platelet reactivity by TEG platelet mapping in patients with minor stroke or TIA.

## RESULTS

### Study participants and baseline characteristics

Among 675 patients enrolled in PRINCE trial, 339 patients were included in TEG subgroup. Baseline characteristics of patients included and excluded in the subgroup was shown in Table 1. The median age of participants included in TEG subgroup was 61 years, and 28.6% of them were women. The index event was minor stroke in 284 patients (83.8%) and TIA in 55 patients (16.2%). Baseline characteristics were also compared between ticagrelor/aspirin group (n**=**170) and clopidogrel/aspirin group (n**=**169) and they were well balanced between two groups (Table 2).

**Table 1:** Baseline characteristics of patients included and excluded in the subgroup.

**Characteristic**	**Included (n=339)**	**Excluded (n=336)**	***P* value**
Age (y)	61.0(55.0-67.0)	61.0(54.0-67.0)	0.43
Female	97(28.6)	84(25.0)	0.29
BMI (kg/m^2^)	24.5(22.5-26.7)	25.0(22.9-27.4)	0.052
Medical history			
Hypertension	196(57.8)	215(64.0)	0.10
Dyslipidemia	23(6.8)	18(5.4)	0.44
Diabetes mellitus	72(21.2)	92(27.4)	0.06
Ischemic stroke	59(17.4)	62(18.5)	0.72
TIA	9 (2.7)	9(2.7)	0.98
Coronary artery disease	13(3.8)	38(11.3)	0.0002
Current smoker	150(44.3)	169(50.3)	0.20
Medication use			
Proton-pump inhibitor	3(0.9)	2(0.6)	0.66
Statin	38(11.2)	28(8.3)	0.21
Aspirin	73(21.5)	73(21.7)	0.95
Clopidogrel	9(2.7)	6(1.8)	0.44
Ticagrelor	0(0.0)	0(0.0)	-
Time to randomization (h)	14.4(7.9-20.8)	13.8(8.4-20.5)	0.94
Qualifying event			0.88
Minor stroke	284(83.8)	280(83.3)	
TIA	55(16.2)	56(16.7)	
SSS-TOAST stroke subtype			0.02
Large-artery atherosclerosis	157(55.3)	147(52.5)	
Cardioaortic embolism	7(2.5)	6(2.1)	
Small-artery occlusion	106(37.3)	107(38.2)	
Other causes	2(0.7)	14(5.0)	
Undetermined causes	12(4.2)	6(2.1)	

Abbreviations: BMI, body mass index; TIA, transient ischemic attack; SSS-TOAST, Stop Stroke Study Trial of Org 10172 in Acute Stroke Treatment.

Data are given as median (interquartile range) and n (%).

**Table 2:** Baseline characteristics of participants stratified by antiplatelet therapy.

**Characteristics**	**Ticagrelor/Aspirin (n=170)**	**Clopidogrel/Aspirin (n=169)**	***P* value**
Age (y)	63.0(55.0-67.0)	61.0(55.0-67.0)	0.32
Female	50(29.4)	47(27.8)	0.74
BMI (kg/m^2^)	24.2(22.6-26.5)	24.6(22.5-26.7)	0.85
Medical history			
Hypertension	101(59.4)	95(56.2)	0.55
Dyslipidemia	13(7.7)	10(5.9)	0.53
Diabetes mellitus	37(21.8)	35(20.7)	0.81
Ischemic stroke	25(14.7)	34(20.1)	0.19
TIA	4(2.4)	5(3.0)	0.73
Coronary artery disease	7(4.1)	6(3.6)	0.79
Current smoker	75(44.1)	75(44.4)	0.71
Medication use			
Proton-pump inhibitor	1(0.6)	2(1.2)	0.56
Statin	22(12.9)	16(9.5)	0.31
Aspirin	42(24.7)	31(18.3)	0.15
Clopidogrel	3(1.8)	6(3.6)	0.31
Ticagrelor	0(0.0)	0(0.0)	
Time to randomization (h)	15.5(8.0-20.8)	13.4(7.9-20.7)	0.50
Qualifying event			
Minor stroke	143(84.1)	141(83.4)	0.86
TIA	27(15.9)	28(16.6)	
SSS-TOAST stroke subtype			0.80
Large-artery atherosclerosis	81(56.6)	76(53.9)	
Cardioaortic embolism	4(2.8)	3(2.1)	
Small-artery occlusion	53(37.1)	53(37.6)	
Other causes	1(0.7)	1(0.7)	
Undetermined causes	4(2.8)	8(5.7)	

Abbreviations: BMI, body mass index; TIA, transient ischemic attack; SSS-TOAST, Stop Stroke Study Trial of Org 10172 in Acute Stroke Treatment.

Data are given as median (interquartile range) and n (%).

### Effect of ticagrelor/aspirin versus clopidogrel/aspirin on platelet reactivity

At 7+2 days, compared with clopidogrel/aspirin group, the proportion of HOPR in ticagrelor/aspirin group was significantly lower [ADP-induced maximum amplitude (MA_ADP_) > 47mm, 4.8% vs 28.1%, *P* < 0.001; ADP inhibition rate (ADP%) < 30%, 4.2% vs 25.5%, *P* < 0.001; arachidonic acid inhibition rate (AA%) < 50%, 9.6% vs 21.0%, *P* = 0.004]. At 90±7 days, similar results were obtained (MA_ADP_ > 47mm, 12.2% vs 30.0%, *P* < 0.001; ADP% < 30%, 6.5% vs 29.3%, *P* < 0.001) (Table 3).

**Table 3:** Effect of ticagrelor/aspirin versus clopidogrel/aspirin on platelet reactivity.

**HOPR**	**Ticagrelor/Aspirin (n=170)**	**Clopidogrel/Aspirin (n=169)**	**RR (95%CI)**	***P* value**
7+2 days				
AA%<50%	16/167(9.6)	35/167 (21.0)	0.46(0.26-0.78)	0.004
ADP%<30%	7/168(4.2)	41/161(25.5)	0.16(0.07-0.33)	<0.001
MA_ADP_>47mm	8/168(4.8)	47/167 (28.1)	0.17(0.08-0.33)	<0.001
90±7 days				
ADP% <30%	10/153(6.5)	44/150(29.3)	0.22(0.11-0.41)	<0.001
MA_ADP_ >47mm	19/156(12.2)	46/154(30.0)	0.41(0.24-0.65)	<0.001

Abbreviations: HOPR, high on-treatment platelet reactivity; AA, arachidonic acid; ADP, adenosine diphosphate; MA, maximum amplitude.

CI, confidence interval; RR, risk ratio. Data are given as n(%).

### Effect of ticagrelor/aspirin versus clopidogrel/aspirin on platelet reactivity stratified by *CYP2C19* LOF allele carrier status

**Figure 1:**
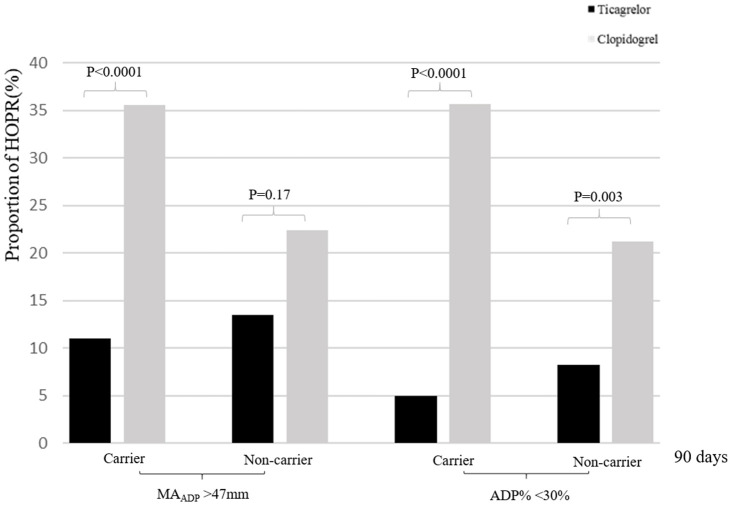
**Proportion of HOPR stratified by dual antiplatelet therapy and *CYP2C19* loss-of-function carrier status at 90±7 days.** HOPR indicates high on-treatment platelet reactivity; ADP, adenosine diphosphate.

In TEG subgroup, 189 patients (55.8%) were *CYP2C19* LOF allele carriers and 150 (44.2%) were non-carriers. At 7+2 days, the proportion of HOPR on aspirin was significantly different between two treatment groups among carriers (AA% < 50%, 7.8% versus 21.9%; *P* = 0.007), but not among non-carriers (AA% < 50%, 11.7% versus 19.7%; *P* = 0.18). The proportion of HOPR on ticagrelor or clopidogrel was significantly lower in ticagrelor/aspirin group versus clopidogrel/aspirin group among carriers and non-carriers. At 90±7 days, compared with clopidogrel/aspirin group, ticagrelor/aspirin group had a lower proportion of HOPR among carriers (MA_ADP_ > 47mm, 11.0% versus 35.6%, *P* < 0.001), but no significant difference was found among non-carriers (MA_ADP_ > 47mm, 13.5% versus 22.4%, *P* = 0.17). However, no significant treatment-by-genotype interactions were found (*P* for interaction = 0.19) (Table 4, Figure 1).

**Table 4:** Effect of ticagrelor/aspirin versus clopidogrel/aspirin on platelet reactivity stratified by *CYP2C19* loss-of-function carrier status.

**HOPR**	**Carrier**					**Non-carrier**				***P* for interaction**
	**Ticagrelor/ Aspirin (n=91)**	**Clopidogrel/Aspirin (n=98)**	**RR (95%CI)**	***P* value**		**Ticagrelor/Aspirin (n=79)**	**Clopidogrel/Aspirin (n=71)**	**RR (95%CI)**	***P* value**	
7+2 days										
AA%<50%	7/90(7.8)	21/96(21.9)	0.36(0.15-0.75)	0.007		9/77(11.7)	14/71(19.7)	0.59(0.26-1.27)	0.18	0.37
ADP%<30%	4/90(4.4)	25/90(27.8)	0.16(0.05-0.39)	<0.0001		3/78(3.9)	16/71(22.5)	0.17(0.04-0.49)	<0.0001	0.94
MA_ADP_ >47mm	3/90(3.3)	29/96(30.2)	0.11 (0.03-0.30)	<0.0001		5/78(6.4)	18/71(25.4)	0.25(0.09-0.60)	0.001	0.27
90±7 days										
ADP% <30%	4/80 (5.0)	30/84(35.7)	0.14(0.04-0.33)	<0.0001		6/73(8.2)	14/66(21.2)	0.39(0.14-0.90)	0.003	0.14
MA_ADP_ >47mm	9/82(11.0)	31/87(35.6)	0.31(0.15-0.58)	<0.0001		10/74(13.5)	15/67(22.4)	0.60(0.28-1.24)	0.17	0.19

Abbreviations: HOPR, high on-treatment platelet reactivity; AA, arachidonic acid; ADP, adenosine diphosphate; MA, maximum amplitude.

CI, confidence interval; RR, risk ratio. Data are given as n (%).

### Effect of ticagrelor/aspirin versus clopidogrel/aspirin on clinical outcomes

At 90±7 days, although there was a relatively lower risk of stroke, composite vascular events and ischemic stroke and a higher risk of any bleeding in ticagrelor/aspirin versus clopidogrel/aspirin group, no significant differences were found in the risk of efficacy and safety outcomes between two groups (Table 5).

**Table 5:** Effect of ticagrelor/aspirin versus clopidogrel/aspirin on clinical outcomes.

**Outcome**	**Ticagrelor/ Aspirin (n=170)**	**Clopidogrel/ Aspirin (n=169)**	**HR (95%CI)**	***P* value**
**Efficacy outcome**				
Stroke	9(5.3)	13(7.7)	0.68(0.29-1.59)	0.37
Composite vascular events	10(5.9)	15(8.9)	0.65(0.29-1.45)	0.29
Ischemic stroke	9(5.3)	12(7.1)	0.74(0.31-1.75)	0.49
**Safety outcome**				
Major bleeding	0(0)	2(1.2)	-	0.15
Any Bleeding	33(19.4)	25(14.8)	1.35(0.80-2.27)	0.26

CI, confidence interval; HR, hazard ratio. Data are given as n (%).

## DISCUSSION

TEG subgroup analysis of the PRINCE trial demonstrated that ticagrelor was superior to clopidogrel in inhibiting platelet reactivity assessed by TEG among patients with acute minor stroke or TIA, particularly in carriers of the *CYP2C19* LOF alleles.

The main analysis of PRINCE trial where platelet reactivity was assessed by VerifyNow P2Y12 assay in 675 patients with acute minor stroke or TIA has made a consistent conclusion with the TEG subgroup analysis [18]: ticagrelor was more efficacious than clopidogrel in inhibiting platelet reactivity. In the study, platelet function was evaluated using TEG platelet mapping assay. Although our results showed that there was no significant difference in the risk of recurrent ischemic stroke between two treatment groups, this may be related to the limited sample size and large trials are needed.

Some studies have showed that TEG has good correlation with light transmission aggregometry and VerifyNow P2Y12 assay in assessing platelet function [19, 29]. Previous studies suggested that MA measured by TEG can predict risk for ischemic events after coronary stenting, which showed that TEG can be effective in monitoring antiplatelet efficacy [20–26]. However, data about TEG parameters in patients with minor stroke or TIA are very limited. A study confirmed that MA_ADP_ >47 mm had good predictive value for recurrent ischemic events after minor stroke or TIA and it has auxiliary effect on clinical decision-making, which enrolled patients with dual antiplatelet therapy of clopidogrel and aspirin [30]. Therefore, we adopted MA_ADP_ >47 mm as the cut-off value to define HOPR for treatment with P2Y12 receptor antagonist in this study. Our study affords more evidence for the application of TEG in assessing the efficacy of antiplatelet therapies in minor stroke or TIA patients.

Ticagrelor is a reversible P2Y12 receptor antagonist, and inhibits platelet reactivity regardless of *CYP2C19* genotypes. The metabolism of clopidogrel is regulated by the cytochrome P450 system (especially *CYP2C19*) and carriers of *CYP2C19* LOF alleles had poor responses to clopidogrel in patients with acute minor stroke or TIA [9, 10]. Our results showed that ticagrelor versus clopidogrel had a lower proportion of HOPR at 90±7 days among carriers, but not among non-carriers. Although interaction between treatment and genotype groups was not significant for the proportion of HOPR, the small sample size might explain this. We speculated that the superiority of ticagrelor over clopidogrel in inhibiting platelet reactivity in carriers of *CYP2C19* LOF alleles might contribute to attenuated clopidogrel metabolism. Carriers of *CYP2C19* LOF alleles might achieve more benefit with ticagrelor plus aspirin than clopidogrel plus aspirin, which indicates that use of ticagrelor instead of clopidogrel might eliminate the need for genetic testing before dual antiplatelet therapy. However, the findings of our study should be evaluated further in large, phase III trials and in different populations.

There were several advantages in our study. First, the design was a pre-specified subgroup analysis of a randomized controlled trial. Second, both platelet reactivity and clinical outcomes were compared between two treatment groups to investigate the effect of ticagrelor versus clopidogrel on platelet function in patients with minor stroke or TIA. There were several limitations. First, TEG platelet mapping was not measured at baseline. However, considering that the study was a subgroup of a randomised controlled trial and baseline characteristics were well balanced between two treatment groups, platelet reactivity at baseline should be balanced. Second, about 9% (n=29) of the patients were lost to follow-up for the evaluation of platelet function at 90 days. However, similar results were observed after assuming that all the missing data were HOPR, or not. Third, baseline characteristics of patients included (n=339) and excluded (n=336) in TEG subgroup were not totally balanced, including the proportions of patients with coronary artery disease and SSS-TOAST stroke subtype. Therefore, the generalization of our results is limited and more randomized trails are needed to confirm them.

In conclusion, ticagrelor plus aspirin was superior to clopidogrel plus aspirin in inhibiting platelet reactivity assessed by TEG in patients with acute minor stroke or TIA, particularly in carriers of the *CYP2C19* LOF alleles. Large randomised controlled trials are needed to confirm our findings.

## MATERIALS AND METHODS

The data supporting the findings of this study are available from the corresponding author upon reasonable request.

### Study population

A pre-specified subgroup analysis of PRINCE trial (Clinicaltrials.gov NCT02506140) was conducted, which was a randomised, prospective, multicenter, open-label, active-controlled, blind-endpoint trial. The rationale and design of PRINCE trial have been described previously [31]. Briefly, the trial randomised patients with acute minor ischemic stroke (National Institutes of Health Stroke Scale score ≤3), or moderate to high risk TIA (ABCD^2^ stroke risk score of ≥ 4 or ≥ 50% stenosis of cervical or intracranial vessels that could account for the presentation) to ticagrelor/aspirin group or clopidogrel/aspirin group within 24 hours of symptoms onset. Patients were excluded from the trial if they had a diagnosis intracranial haemorrhage, acute coronary syndrome, or other pathology that could account for the neurological symptoms; had a modified Rankin scale score of more than 2 at randomization; or had a contraindication to ticagrelor, clopidogrel, or aspirin. The flow diagram of PRINCE trial has been previously presented [18]. A total of 339 participants from 12 centers who assessed platelet function by TEG platelet mapping were included in our subgroup analysis.

Participants received ticagrelor (180 mg on day 1, followed by 90 mg twice daily on days 2–90) or clopidogrel (300 mg on day 1, followed by 75 mg daily on days 2–90) with an aspirin background using (300 mg on day 1, followed by 100 mg daily on days 2–21). In brief, the whole antiplatelet treatment lasted for 90 days and the duration time of dual antiplatelet therapy was 21 days. Patient enrolment began in China in August 2015, and patient follow-up was completed by June 2017. The study’s protocol was approved by the participating hospitals’ ethics committees. All of the participants provided written informed consent.

### Measurement of platelet reactivity

Platelet reactivity was assessed by the TEG platelet mapping (TEG 5000; Haemonetics, Braintree, Massachusetts, USA) at 7+2 days and 90±7 days after randomization. Blood for platelet function evaluation was sampled and assessed between 2 and 4 hours after the morning maintenance dose of antiplatelet drugs. TEG was performed according to manufacturers’ instructions and used four channels to detect effects of antiplatelet therapy with AA and ADP activators [32]. MA represents the maximal clot strength. MA_ADP_ is the ADP-induced clot strength, MA_AA_ is the AA-induced clot strength, MA_fibrin_ is the activator-induced clot strength (measurement of fibrin contribution), and MA_thrombin_ is the thrombin-induced clot strength. The percentage of platelet inhibition by ticagrelor or clopidogrel was computed as the contribution of ADP-stimulated platelets to maximal clot strength: ADP%=[(MA_thrombin_- MA_ADP_)/(MA_thrombin_-MA_fibrin_)]×100%. The percentage of platelet inhibition by aspirin was computed as the contribution of AA-stimulated platelets to maximal clot strength: AA%=[(MA_thrombin_- MA_AA_)/(MA_thrombin_-MA_fibrin_)]×100%. AA%<50% can be considered as HOPR on aspirin and ADP%<30% or MA_ADP_>47mm can be considered as HOPR on ticagrelor or clopidogrel [23, 33]. Platelet function testing was conducted according to a standardized procedure manual in each study center by qualified personnel who were blinded to treatment allocation. Both the investigators and patients were aware of the study drug assignment, but were blinded to platelet reactivity data until the end of the trial.

### Outcomes assessment

The study’s primary outcome was the proportion of patients with HOPR (defined as MA_ADP_ >47mm) at 90±7 days [28, 30, 33]. Secondary outcomes were clinical outcomes at 90±7 days. Efficacy outcomes included any stroke (ischemic or hemorrhagic) and composite vascular events (stroke, TIA, myocardial infarction, or vascular death) at 90 days. Safety outcomes included bleeding events which were defined according to PLATO criteria [34].

### *CYP2C19* genotyping

Blood samples were collected and shipped via cold-chain transportation from each center to Beijing Tiantan Hospital and stored at -80 °C. Three *CYP2C19* single-nucleotide polymorphisms (SNPs) were assessed, including *2 (681G>A, dbSNP rs4244285), *3 (636G>A, dbSNP rs4986893) and *17 (−806C>T, dbSNP rs12248560). Genotyping was performed on the Sequenom MassARRAY iPLEX platform (Sequenom, San Diego, California, USA) and Sanger sequencing (ABI 3500 Genetic Analyzer, Applied Biosystems, Foster City, CA, USA) if the results were otherwise inconclusive. The call rate of each SNP was >98.5%. Carriers of LOF alleles were defined as patients with at least one LOF allele (*2 or *3), including the genotypes *1/*2, *1/*3, *2/*2, *2/*3, *3/*3, *2/*17, or *3/*17. Non-carriers were defined as patients with no LOF alleles (*2 or *3), including the genotypes *1/*1, *1/*17, or *17/*17.

### Statistical analysis

Continuous variables were presented as mean with standard deviation, or median with interquartile range, and categorical variables as percentages. Baseline characteristics were compared between ticagrelor/aspirin group and clopidogrel/aspirin group, using the Student’s t-test or Wilcoxon test for continuous variables, and the χ^2^ test for categorical variables. We compared the proportion of HOPR between two treatment groups and reported it as a risk ratio (RR) with 95% confidence intervals (CI). The differences in the risks of stroke, composite events, ischemic stroke and bleeding events during the 90 day follow-up were assessed by Cox proportional hazards regression, and were reported as hazard ratios (HR) with 95% CI. We assessed whether the treatment effect differed by testing the treatment-by-genotype interaction effect in genmod models for the proportion of HOPR.

Two-sided *P* values <0.05 were considered statistically significant. All analyses were performed using SAS 9.4 (SAS Institute, Cary, North Carolina).
